# Choice of contraceptive methods in public and private facilities in rural India

**DOI:** 10.1186/s12913-019-4249-0

**Published:** 2019-06-25

**Authors:** Arupendra Mozumdar, Vandana Gautam, Abhishek Gautam, Arnab Dey, Uttamacharya, Ruhi Saith, Pranita Achyut, Abhishek Kumar, Kumudha Aruldas, Amit Chakraverty, Dinesh Agarwal, Ravi Verma, Priya Nanda, Suneeta Krishnan, Niranjan Saggurti

**Affiliations:** 1 Population Council, New Delhi, India; 2 Oxford Policy Management, New Delhi, India; 3 International Center for Research on Women, New Delhi, India; 4 Sambodhi Research and Communications Private Limited, Noida, Uttar Pradesh India; 5 IPE Global, New Delhi, India; 6 Bill and Melinda Gates Foundation, New Delhi, India

**Keywords:** Quality of care, Method choice, Family planning, India

## Abstract

**Background:**

Client-centric quality of care (QoC) in family planning (FP) services are imperative for contraceptive method adoption and continuation. Less is known about the choice of contraceptive method in India beyond responses to the three common questions regarding method information, asked in demographic and health surveys. This study argues for appropriate measurement of method choice and assesses its levels and correlates in rural India.

**Methods:**

A cross-sectional study was conducted with new acceptors of family planning method (*N* = 454) recruited from public and private health facilities in rural Bihar and Uttar Pradesh, the two most populous states in India. The key quality of care indicator ‘method choice’ was assessed using four key questions from client-provider interactions that help in making a choice about a particular method: (1) whether the provider asked the client about their preferred method, (2) whether the provider told the client about at least one additional method, (3) whether the client received information without any single method being promoted by the provider, and (4) client’s perception about receipt of method choice. The definition of method choice in this study included women who responded “yes” to all four questions in the survey. The relationship between contraceptive communication and receipt of method choice was assessed using logistic regression analyses, after adjusting for socio-demographic characteristics of the respondents.

**Results:**

Although 62% of clients responded to a global question and reported that they received the method of their choice, only 28% received it based on responses about client-provider interactions. Receipt of the information on side-effects of the selected method (Adjusted Odds Ratio [AOR]: 7.4, 95% Confidence Interval [CI]: 3.96–13.86) and facility readiness to provide a range of contraceptive choice (AOR: 2.67, 95% CI: 1.48–4.83) were significantly associated with receipt of method choice.

**Conclusions:**

Findings demonstrated that women’s choice of contraceptive could be improved in rural India if providers give full information prior to and during the acceptance of a method and if facilities are equipped to provide a range of choice of contraceptive methods.

## Background

With India’s FP2020 commitment to provide 48 million additional women with modern contraceptives [1] and the anticipated increase in the number of people receiving contraceptive services, maintaining quality of care (QoC) is critical both for contraceptive uptake and continuation [2–6]. Family planning impact studies from Bangladesh, Senegal, and Tanzania showed that women’s contraceptive use was higher in areas where clients felt that they were receiving good care [7–13].

Earlier studies on QoC from India mostly focused on three indicators of method information index (MII), which includes whether the provider informed the client about the side-effects of selected methods; whether the provider explained how to manage the side-effects of the selected method, if experienced; and whether the provider provided information about other FP methods [14]. Recent evidence from India showed that 47% of the women were told about the side-effects of the selected method; 39% of women were told how to manage side-effects, if experienced; and 54% of women were ever told about other FP methods that could be used [15].

In the absence of any medical contraindication, the selection of an FP method by women was often conceptualized as an autonomous decision [16]. The studies from low- and middle-income countries on decision-making were conducted through different research on methodologies [17, 18], including randomized controlled trial [19] and evaluation of counseling tools [20]. The findings of all these studies suggested that giving women the information and choice increases their adoption of contraceptives. Cross-sectional studies in India also demonstrated a positive association between the use of contraceptives with receipt of FP advice from health workers [21]. However, the literature from India mostly described the QoC related to the method that the client already selected and was using but lacks studies on the decision- making process on contraceptive and QoC in FP from the clients’ perspective.

The receipt of the method-choice is one of the indicators of client satisfaction for QoC studies of FP services. In low- and middle-income countries like India, however, poor and uneducated women often report that they received their FP method choice, but the selection of the method was often not their decision [22, 23]. RamaRao et al. [24] assessed method choice based on a set of four indicators of quality of FP services: (1) whether the provider asked which method the client preferred, (2) whether the provider told about at least one additional method besides the method adopted, (3) whether the client received information without any single method being promoted by the provider, and (4) whether the provider gave the client their method choice. If the answer to all four questions is “yes,” the client’s choice of method could be considered a result of her decision–free of any provider bias–and therefore, could be a better indicator of client satisfaction with FP service. This forms the basis of this article, which examines the receipt of method choice of those attending public and private health clinics in rural areas of two large north Indian states.

## Methods

### Study setting

The study was conducted in selected health facilities in Bihar and Uttar Pradesh (UP), the two most populous states of India, with a total population of more than 300 million. Both states are also high-focus states areas for FP because of prevailing high unmet need (21% in Bihar and 18% in UP), high fertility (total fertility rate of 3.4 in Bihar and 2.7 in UP), and low use of modern contraceptive methods (23% in Bihar and 32% in UP) [15]. Historically, the use of modern contraceptive use has remained low in these two states [15, 25]. The method-mix in these two states are different; the percentage of female sterilization among users of any modern method in Bihar is 89% and in UP is 55%. The public sector remains the main source of modern contraceptives (63% for Bihar and 54% for UP) [15].

### Data

The study used baseline data conducted as part of a comprehensive QoC study for FP services in public health facilities in Bihar, and public and private health facilities in UP. The facilities were selected using three stratified random sampling strategies for three types of health facilities: public health facilities in Bihar, public health facilities in UP, and private health facilities in UP. The public health facilities that were classified to receive an intervention or not in Bihar were divided into three strata: district hospitals (DH), community health centers (CHC), and primary health centers (PHC). The number of facilities selected in each stratum was determined by the relative proportion of each type of facility from the list of intervention and non-intervention facilities. Finally, from each of the strata, facilities were selected using probability proportionate to size (PPS) sampling based on numbers of Mini-Lap sterilization (chosen as a proxy to the volume of client load) procedures conducted during the year preceding the survey. However, all the study facilities had short-acting and/or long-acting reversible contraceptive methods on the day of data collection.

For public health facilities in UP, within each district, the health facilities were stratified into two groups: DH and CHC. Of the total sample of facilities, 50% were taken from the program high priority districts (HPDs), and the other 50% were taken from the non-high-priority districts. Health facilities (DH and CHC) within each district were selected using systematic PPS sampling based on numbers of sterilization procedures conducted during the year preceding the survey. The sterilization procedures numbers were chosen to get to enough numbers of new acceptors of family planning. Similar to the public health facilities in Bihar, all selected study facilities in UP had short-acting and/or long-acting reversible contraceptive methods on the day of data collection.

The private health facilities of UP were selected from the list of all private facilities participating in social franchises of Population Services International (PSI) and Hindustan Latex Family Planning Promotion Trust (HLFPPT). Social franchises of PSI are present in 10 districts, and of HLFPPT in 17 districts of UP. The districts were arranged by their modern contraceptive prevalence rate as estimated in Annual Health Survey 2012–13 [26]. Fourteen districts, i.e., seven districts for each of the social franchise networks were selected as study sites using systematic random sampling. Within each district, all clinics under social franchise with a load of 20 or more FP clients per month were included in the sampling frame.

Clients of all three types of facilities (irrespective of the type of contraceptive method they received) were interviewed at the point of exit. The clients were asked about their experiences with FP services in the facility. Specifically, each client was asked a set of questions about the information received about contraceptive methods, respectful care, and their willingness to follow up or return to the facility as a measure of the level of satisfaction with the FP services received in the overall quality of care study. Data chosen for this study included only the clients who answered all the questions across three types of facilities. In total, 454 clients were interviewed from 187 health facilities. The sector-wise break-up of the clients was 158 clients from 61 public health facilities of Bihar, 221 clients of 88 public health facilities of UP, and 75 clients of 38 private health facilities of UP. Of the 158 clients from Bihar public health facilities, 94% were sterilization acceptors, and remaining were acceptors of long-acting reversible methods, or short-acting methods. Of the clients from UP public health facilities, 48% were sterilization acceptors, 30% were acceptors of long-acting reversible methods, and 22% were acceptors of short-acting methods. Of the 75 clients from UP private health facilities, 8% were sterilization acceptors, 91% were acceptors of long-acting reversible contraceptive method, and 1% were acceptors of short-acting methods. The youngest participant was 19 years old.

Three separate research organizations collected the data for this study under a partnership, and standardized and comparable questions were used in data-collection tools. Except for female sterilization clients, all those coming to the facility for FP services on a typical day who agreed to participate in the study were interviewed at the point of exit from the facility. The sterilization clients were interviewed at home, 15 to 30 days after the procedure, because they left the facility on the same day and it was difficult to conduct an exit interview after the sterilization procedure. Information from the sterilized clients was collected through face-to-face interviews in UP and through telephone in Bihar.

Apart from the regular offering, the public health system of Bihar and Uttar Pradesh often offer “fixed-day-services” for family planning. The number of clients served on those “fixed-days” are higher than on a typical day. We expected that the quality of care in those “fixed-days” would be worse than a typical day due to higher client-load, therefore, we conducted the client exit interviews on a typical day, also we kept a similar setting of data collection in public facilities compared to the setting of private facilities. Interviews were conducted by trained female investigators using a structured set of questions in the Hindi language–the local language of these two states. The study design for this analysis used a common set of questions prepared by researchers of all three organizations that collected the data.

#### Outcome indicator: method choice

The outcome indicator in this study was the receipt of method choice among FP clients. This indicator was calculated using responses to four questions from the client exit interview: whether the provider asked about method preference, whether the client was told about at least one additional method, whether the client was not promoted to adopt any one FP method by the provider, and whether the client reported that she received her method choice. Women who answered “yes” to all four questions were considered to have received their method choice.

#### Predictor variables

As existing literature suggests that one element of QoC may affect the other element, a set of QoC process indicators were considered as the predictor variables. These indicators were adopted using the situation analysis approach proposed by Miller et al. [8]. The categories of the predictor variables selected are given below.

##### Facility readiness to provide a method

Both public and private health facilities were assessed for their readiness, on the day of the survey, to provide each of the FP methods, available in the public health system of the state, such as, female sterilization using both laparoscopy and mini-lap procedure, intra-uterine contraceptive device (IUCD), condoms, and oral contraceptive pills. No other contraceptive methods were available in the public sector in India at the time of this study. Each of the selected facilities was assessed using a facility audit checklist that collected data on the basic infrastructure (e.g., availability of water and electricity); the presence of trained staff; availability of drugs, equipment, and supplies required to provide clinical FP services; and availability of commodities of nonclinical FP methods.

Based on the facility readiness of the type of methods, each facility was categorized into one of the four groups: facilities ready to provide only short-acting methods (condoms and pills); facilities ready to provide short-acting methods and the long-acting reversible method (IUCD); facilities ready to provide permanent methods (female sterilization) and either the long-acting reversible method or short-acting methods; and facilities ready to provide all three types of FP methods: permanent, long-acting reversible, and short-acting.

##### Client-provider interaction

The indicators for information given to clients included the variables related to the discussions or information exchange, that the client had, with the family planning service provider. Apart from the four indicators mentioned as a part of method choice, five additional indicators on client-provider interaction were considered: provider told client about the side-effects of the method, provider told client how to manage side-effects if experienced, provider told client the results of tests and examinations, provider encouraged the client to ask questions, and client felt respected by the provider’s behavior.

#### Background characteristics of the clients

Background characteristics of the clients were also considered as predictors. Categories are: age groups of clients (less than 25 years, 25–29 years, 30–34 years, and 35 years and above), client belongs to social group (scheduled castes or scheduled tribes, other backward classes, and general caste), client’s education status (no education or completed less than 5th standard, completed 5th to 9th standard, and completed 10th standard or higher), education status of client’s husband (no education or completed less than 5th standard, completed 5th to 9th standard, and completed 10th standard or higher), and number of living children the woman has (no child, one child, two children, three children, four or more children).

### Statistical analysis

Univariate analyses were used to understand the background characteristics of the clients. The differences in QoC indicators by receipt of method choice, or not, were compared using chi-square tests. Multivariate regression analyses were used to identify the factors associated with the method choice.

To identify the factors associated with the receipt of method choice, binary logistic regressions were performed. In these analyses, the dependent variables were binary variables, where the receipt of method choice was coded as “1,” and “0” otherwise.

Two sets of logistic regressions were used: Model 1a-e using each of the QoC indicators and facility readiness as factors, and Model 2 using all QoC indicators and facility readiness together. Since receipt of information about side effects for the selected method and receipt of the information on how to manage side-effects were highly correlated, the second variable was dropped from the regression analyses. Both the models were adjusted for clients’ background characteristics as confounders. Results obtained from the regression analyses were presented as adjusted odds ratios (AORs) along with the 95% confidence interval (95% CI). All these analyses were done using Stata (version 13) statistical software.

## Results

### Background characteristics of the clients

About half of the clients (52%) aged 25–29 years (Table 1). More than half of the clients (57%) belonged to other backward classes and 27% of the clients belonged to scheduled caste or scheduled tribe families. About half of the clients (46%) had no education or completed less than 5th standard of schooling. About three-fifths (57%) of the clients had three or more children.

**Table 1:** Distribution of the clients across selected sociodemographic characteristics

Background characteristics (*N* = 454)	%
Age of the client	
Less than 25 years	18.2
25–29 years	51.7
30–34 years	22.2
35 years or above	8.0
Social groups	
Scheduled castes/tribes	26.9
Other backward classes	57.3
General	15.9
Client’s education status	
No education/lower than 5th standard	46.0
5th–9th standard	33.0
10th standard and higher	20.9
Client’s husband’s education status	
No education/lower than 5th standard	30.7
5th–9th standard	25.8
10th standard and higher	43.5
Parity	
No child	0.7
1 child	9.9
2 children	32.2
3 children	29.1
4 children or more	28.2

### Quality of care

Quality of FP services as reported by the clients in the study area is presented in Table 2. More than three-fifths (62%) of clients reported that they received their method choice. However, only about half the clients reported that they were told about the side-effects (56%), how to manage side-effects if experienced (55%), the results of tests and examinations done (48%), and were encouraged to ask questions (54%). About three-fourths (73%) of clients reported that the provider asked them about their method preference, 75% of clients reported that the provider gave information without strongly encouraging the client to adopt any one method, and 65% of clients reported that the provider told them about other FP methods than the selected method.

**Table 2:** Percentage of clients reported receipt of quality of care in family planning services

Quality of care indicators (*N* = 454)	n	%
Provider asked about preferences of client	331	72.9
Provider told client about other methods	295	65.0
Provider gave information without promoting any single method	342	75.3
Provider told client about side-effects of the method	254	56.0
Provider told client how to manage side-effects, if experienced	248	54.6
Client was told the results of tests and examinations	219	48.2
Provider encouraged client to ask questions	245	54.0
Client reported receiving method of her choice	279	61.5
Client felt respected	395	87.0

To examine how many clients were informed about different methods without provider bias, and could therefore be considered as having received free (of pressure) and informed “method choice,” we examined the percentage of clients who were asked about their method preference before they chose a method, told about methods other than the selected method, and not coerced to accept a particular method.

#### FP method choice

**Fig. 1:**
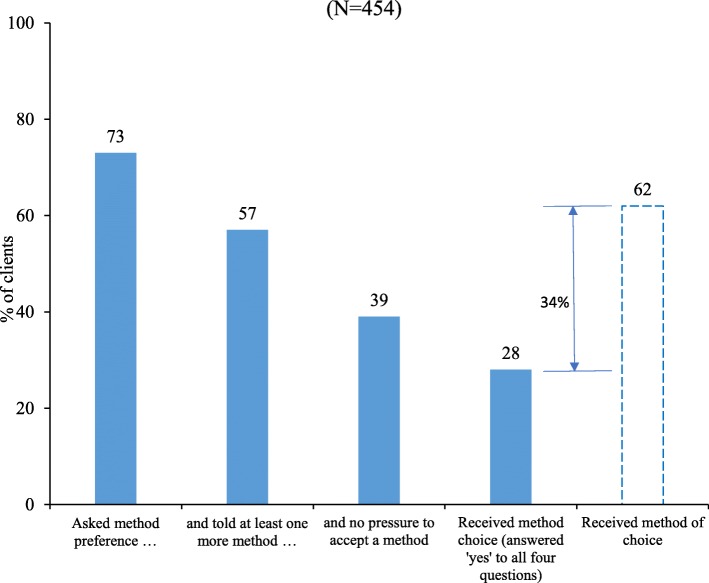
Information cascade for receipt of method choice, compared with reporting of receipt of method of choice using single-question response

Figure 1 shows the proportion of clients who received method choice, as defined in this study, and comparison of clients’ reporting receipt of the method of choice in response to a global question. The results show that 73% of clients were asked about their method preference during counseling. About 57% of clients were asked about their method preference and were also informed about different FP methods. About two-fifths (39%) of clients who were asked about method preference were also informed about other FP methods without the provider encouraging a particular method. In all, 28% of clients reported receipt of method choice by answering “yes” to all four questions. This is a considerable drop from the proportion of clients (62%) who reported that they received method choice by answering just one question on method choice in the questionnaire. If we consider method choice as a score by adding the number of “yes” responses, the score ranges from 0 to 4 and the mean ± SD of the score was 2.7 ± 1.0.

#### Association between receipt of method choice and other QoC indicators

Table 3 presents a comparison of receipt of the information on side-effects among clients who did or did not receive the method choice. The findings show the clients who did not receive method choice also received significantly less information about side-effects. For example, 87% of the clients who received method choice by the study definition also received information about the side-effects. However, only 44% of the clients, who did not receive method choice, received information about side-effects. Similarly, among the clients who were not informed about FP methods, a significantly smaller proportion received other elements of care than the clients who received method choice. Those other elements of care in contraceptive services provision were receipt of the information on how to manage side-effects, receipt of information about test results and examinations, encouragement from providers to ask questions, and feeling of being respected.

**Table 3:** Comparison of quality of care in family planning services between clients who received and who did not receive method choice

Quality of care indicators	Did not receive method choice	Received method choice
	*N* = 328	*N* = 126
Provider told client about the side-effects of the method*	43.9	87.3
Provider told client how to manage side-effects, if experienced*	43.9	82.5
Provider told results of tests and examinations	47.3	50.8
Provider encouraged client to ask questions*	48.5	68.3
Client felt respected*	84.5	93.7

* Significant difference between the two groups of clients *p* < 0.05, Chi-square test

#### Determinants of receipt of method choice

Table 4 shows the results of logistic regressions examining the correlates of receipt of method choice. The results of multivariate logistic regression suggest that clients of facilities that were ready to provide both long-acting reversible and short-acting methods have more than two and half times higher odds of receiving method choice (AOR = 2.67, 95% CI 1.48–4.83) than the clients of facilities having only short-acting methods. Similarly, clients who received services from the facilities ready to provide all three methods (permanent methods, long-acting reversible methods, and short-acting methods) also have higher odds of receiving method choice (AOR = 1.45, 95% CI 0.62–3.42) than the clients of facilities having only short-acting methods. Women’s receipt of method choice is also higher if they were given information about the side-effects of the selected method (AOR = 7.40, 95% CI 3.96–13.86) than if they were not given information about side-effects.

**Table 4:** Odds ratio obtained from binary logistic regression analysis showing the determinants of receipt of method choice

	Model-1					Model-2
	(a)	(b)	(c)	(d)	(e)	
	AOR (95% CI)	AOR (95% CI)	AOR (95% CI)	AOR (95% CI)	AOR (95% CI)	AOR (95% CI)
Factors						
Facility readiness for type of methods						
Only for short-acting methods	Ref.					Ref.
Long-acting reversible and short- acting methods	**3.63 (2.10–6.24)**					**2.67 (1.48–4.83)**
Permanent method and any other method	0.62 (0.27–1.41)					0.55 (0.23–1.35)
Permanent, long-acting reversible and short-acting methods	**2.45 (1.08–5.55)**					1.45 (0.62–3.42)
Provider told client about the side-effects of the method		**9.52 (5.30–17.11)**				**7.40 (3.96–13.86)**
Provider told results of tests and examinations			1.22 (0.80–1.87)			0.94 (0.57–1.54)
Provider encouraged client to ask questions				**2.41 (1.54–3.79)**		1.20 (0.71–2.01)
Client felt respected					**2.58 (1.17–5.66)**	1.78 (0.75–4.18)
Confounders						
Age groups						
Less than 25 years	2.01 (0.66–6.12)	1.97 (0.60–6.45)	1.95 (0.67–5.70)	2.37 (0.79–7.12)	1.93 (0.66–5.64)	1.87 (0.54–6.44)
25–29 years	1.67 (0.65–4.33)	2.32 (0.85–6.30)	2.02 (0.80–5.06)	2.52 (0.98–6.48)	2.04 (0.82–5.13)	1.94 (0.69–5.48)
30–34 years	1.73 (0.65–4.64)	1.75 (0.63–4.89)	1.93 (0.75–5.01)	2.13 (0.81–5.59)	1.88 (0.73–4.87)	1.46 (0.50–4.26)
35 years or above	Ref.	Ref.	Ref.	Ref.	Ref.	Ref.
Social groups						
Scheduled castes/tribes	0.70 (0.33–1.49)	1.09 (0.50–2.39)	0.71 (0.34–1.47)	0.84 (0.40–1.75)	0.73 (0.35–1.51)	0.99 (0.44–2.26)
Other backward classes	1.05 (0.54–2.02)	1.36 (0.68–2.70)	1.15 (0.61–2.15)	1.24 (0.65–2.35)	1.14 (0.60–2.14)	1.17 (0.57–2.38)
General	Ref.	Ref.	Ref.	Ref.	Ref.	Ref.
Client’s education status						
No education/lower than 5th standard	Ref.	Ref.	Ref.	Ref.	Ref.	Ref.
5th–9th standard	1.17 (0.71–1.94)	1.14 (0.67–1.93)	1.26 (0.78–2.03)	1.19 (0.73–1.94)	1.19 (0.74–1.91)	1.11 (0.64–1.94)
10th standard and higher	**0.39 (0.19–0.80)**	0.55 (0.28–1.10)	0.55 (0.29–1.04)	0.56 (0.29–1.07)	0.54 (0.28–1.02)	**0.42 (0.20–0.89)**
Parity						
No child/1 child	0.94 (0.36–2.48)	0.92 (0.33–2.55)	1.24 (0.50–3.07)	1.12 (0.44–2.84)	1.26 (0.51–3.14)	0.78 (0.27–2.27)
2 children	0.83 (0.44–1.57)	0.67 (0.34–1.33)	0.90 (0.49–1.64)	0.79 (0.43–1.46)	0.88 (0.48–1.60)	0.63 (0.31–1.30)
3 children	0.80 (0.44–1.47)	0.63 (0.33–1.19)	0.80 (0.45–1.43)	0.70 (0.39–1.28)	0.80 (0.45–1.42)	0.62 (0.31–1.21)
4 children or more	Ref.	Ref.	Ref.	Ref.	Ref.	Ref.

Note: *AOR* Adjusted Odds Ratio, *CI* Confidence Interval, *Ref.* Reference category, AORs in bold are significant at *p* < 0.05

Model 1a-e: Effect of each of the factors was estimated separately for (a) facility readiness for type of methods, (b) provider told client about the side-effects of the method, (c) provider told results of tests and examinations, (d) provider encouraged client to ask questions, and (e) client felt respected. All models were adjusted for client’s age, parity, education, and social group

Model 2: Effect of all factors were estimated together and adjusted for client’s age, parity, education, and social group

## Discussion

Study findings highlight that there is a huge difference between those reporting receipt of method choice via a single question versus when it is examined in combination with the extent of information exchange during the selection of method choice. The receipt of method choice with complete information is far from universal. The results further indicate that method choice linked to provider-client interaction is more likely to happen if the facilities are equipped to provide various contraceptive methods. This finding is relevant given findings from previous research [27] that have highlighted the likelihood of providers giving information about the methods that are available in the facilities. Further, the number of women reporting receipt of method choice is likely to be high if they are informed about the side-effects of the method.

For the first time in India, this study explored multiple aspects of receipt of method choice controlling for facility readiness and other elements of service provision, therefore, findings from this study could not be compared with other studies because of the dearth of literature on receipt of method choice within the country. RamaRao et al. [24] reported a higher mean score of method choice (3.5) in the Philippines than this study (2.7), however, RamaRao et al. [24] did not report how many clients scored “4” in method choice score, i.e. who answered “yes” to all four questions. Therefore, the findings of this study could serve as the benchmark for measurement on the method choice for future studies in similar settings. Overall, the study revealed that when women were asked about their method preference, and received information about other available methods, side-effects of the chosen method, and the test and examination results, they were more likely to report that they chose the FP method by themselves.

Although less than one-third of the clients reported they received method choice, most clients (87%), regardless of whether they received method choice or not, felt respected during FP services, and most clients (more than 75%) also reported that the provider did not encourage any one method. This reflects the Government of India’s commitment to expand and give choice to women for contraception [1]. The findings in this study also highlight the utility of comprehensively measuring the information exchange between provider and client for understanding the receipt of method choice, which is one of the critical indicators of quality of care.

Although the findings have several implications to measurement and utility of assessing client’s method choice as part of the quality of care studies, the results may be interpreted in the light of certain limitations. Firstly, most women included in this study were younger than 30 years of age, belonged to other backward classes and the scheduled castes and scheduled tribes, therefore, these findings reflect the contraceptive service-related care for young women from marginalized sections of the society seeking services from public (mostly) and private health sectors in the study geographies. Secondly, the number of women that could be recruited from each site was limited due to the low uptake of family planning services by people and the type of methodology adopted in the study. We have carried out clients’ exit interview on a typical day leading to lower numbers, resulting from very low uptake of reversible contraceptive methods. Future prospective research is needed to examine the effects of method choice on the continuation of reversible methods or switching to other methods. Thirdly, the data represents reporting from clients, the reporting may have some personal biases. Some may argue that it would be better to observe the information exchange between the providers and clients with a structured checklist for appropriate assessment of quality in the provision of contraceptive services. On the other hand, the observation data of the provider-client interaction may have some Hawthorne effect (alternation of the natural behavior of the subject of a study due to their awareness of being observed) and that may overestimate the level of quality of care. Alternatively, a “mystery client” methodology for quality of care in reversible contraceptive methods provision may provide realities of provider-client interaction.

While the client-centric contraceptive services continue to be the focus of the FP program, study results also highlight the differences in usual response about the receipt of method choice and whether women received information while making a mention of receipt of method choice or not. It poses both programmatic and rights-based questions. The programmatic approach may be that women are coming to a facility for a particular method, and given that context, how relevant it is for the providers to talk about other methods. The rights-based approach recommends that women should have the right to information from providers at the facility and to make an autonomous and fully informed decision about their preferred method of contraception, irrespective of their preparedness before coming to a facility. Autonomy and full information require that any counseling, advice, or information provided to the women should be done by appropriately trained personnel using language and methods that can be easily understood by the client. Therefore, health-care providers have the responsibility to convey accurate information and noncoercive counseling, and to facilitate fully informed decision-making [28, 29]. The rights-based approach is critical given that most of the women in rural India and other developing countries are illiterate and belong to poor economic households, therefore, women should get complete information at the point of service delivery by the providers, irrespective of the method they may ask for at the beginning of their consultation. This has implications to change thinking in program efforts to promote the continuation of method use, because improved method choice leads to higher continuation rate of contraceptive use [24].

Historically, a skewed method mix indicates either provider bias in the system or user preferences, or both [30, 31]. The method-mix of the two states under study, especially Bihar, are heavily skewed toward female sterilization. Given that clients in these two states are predominantly adopting only one method, it is necessary to study in-depth how provider bias could influence the contraceptive decision-making of the women and therefore their contraceptive use.

## Conclusions

Quality of care in FP reinforces the client’s rights to information, choice, and the quality of interaction with the provider. Quality FP services lead to client satisfaction and that increases the chance of continuing contraceptive use if not continuing the same method. QoC also enhances the job satisfaction of the provider and motivates the provider to deliver better services [32]. But, encouraging the providers to provide sufficient information irrespective of clients’ prior knowledge on methods may need emphasis in the current family planning program.

## Data Availability

Data would be made available from the corresponding author on reasonable request.

## Abbreviations

AOR: Adjusted odds ratio
CHC: Community health center
CI: Confidence interval
DH: District hospital
FP: Family planning
HLFPPT: Hindustan Latex Family Planning Promotion Trust
HPD: High priority district
IUCD: Intra-uterine contraceptive device
MII: Method information index
PHC: Primary health center
PPS: Probability proportionate to size
PSI: Population Services International
QoC: Quality of care
SD: Standard deviation
UP: Uttar Pradesh
